# 

**Published:** 1879-01

**Authors:** 


### Chicago Medical Journal

AND

### EXAMINER.

VOL. XXXVIII.—JANUARY, 1879.—NO. 1.

### To our Readers and Correspondents.

Beginning with Vol. XXXVII., July, 1878, the CHICAGO MEDICAL JOURNAL AND EXAMINER prints all records of length and weight in terms of the Metric System, and all records of temperature in degrees of the Centigrade Scale. The Metric System, legalized in the United States and Great Britain, is extensively or exclusively employed by other civilized nations, and has thus become an essential part of the international language of science. It is recommended for adoption to the profession in this country by the American Medical Association and other scientific bodies. To-day no physician can afford to be ignorant of its value, its simplicity and the meaning of its terms.

The subjoined tables and scales, which have been kindly prepared for us by Prof. W. S. Haines, will be continuously reproduced in subsequent numbers of this journal, for the ready reference of our readers and correspondents.

### METRIC MEASURES OF LENGTH.

| | | | |
|---|---|---|---|
| Millimeter | 0.001 | of a Meter | 0.03937 inches. |
| Centimeter | 0.01 | " " | 0.39370 " |
| Decimeter | 0.1 | " " | 3.93707 " |
| **Meter** | **1.** | **Meter** | **39.37079** " |
| Decameter | 10. | Meters | 393.70790 " |
| Hectometer | 100. | " | 3937.07900 " |
| Kilometer | 1000. | " | 39370.79000 " |

### METRICAL WEIGHTS.

| | | | |
|---|---|---|---|
| Milligram | 0.001 | of a Gram | 0.015 grains. |
| Centigram | 0.01 | " " | 0.154 " |
| Decigram | 0.1 | " " | 1.543 " |
| **Gram** | **1.** | **Gram** | **15.432** " |
| Decagram | 10. | Grams | 154.323 " |
| Hectogram | 100. | " | 1543.234 " |
| Kilogram | 1000. | " | 15434.348 " |

☞ The United States "nickel" five cent piece weighs five grams, and is two centimeters in diameter.

### APPROXIMATE EQUIVALENT OF METRICAL WEIGHTS.

*For Rapid Reference.*

| *Milligrams.* | *Grains.* | *Decigrams.* | *Grains.* |
|---|---|---|---|
| 1 (written 0.001 or \|001)* | $\frac{1}{65}$ | 1 (written 0.1 or \|1) | $1\frac{1}{2}$ |
| 2 | $\frac{1}{32}$ | 2 | 3 |
| 3 | | 3 | $4\frac{1}{2}$ |
| 4 | $\frac{1}{16}$ | 4 | 6 |
| 5 | $\frac{1}{13}$ | 5 | $7\frac{1}{2}$ |
| 6 | $\frac{1}{11}$ | 6 | 9 |
| 7 | $\frac{1}{9}$ | 7 | 11 |
| 8 | $\frac{1}{8}$ | 8 | $12\frac{1}{2}$ |
| 9 | $\frac{1}{7}$ | 9 | 14 |

| *Centigrams.* | *Grains.* | *Grams.* | *Grains.* |
|---|---|---|---|
| 1 (written 0.01 or \|01) | $\frac{1}{6}$ | 1 (written 1. or 1\|) | 15 |
| 2 | $\frac{1}{3}$ | 2 | 30 |
| 3 | $\frac{6}{13}$ | 3 | 46 |
| 4 | $\frac{7}{11}$ | 4 | 61 |
| 5 | $\frac{3}{4}$ | 5 | 77 |
| 6 | $\frac{9}{10}$ | 6 | 92 |
| 7 | 1 | 7 | 108 |
| 8 | $1\frac{1}{4}$ | 8 | 123 |
| 9 | $1\frac{1}{3}$ | 9 | 139 |

A Kilogram=$2\frac{1}{5}$ lbs. Avoirdupois.

* The decimal *line* instead of points makes errors impossible.

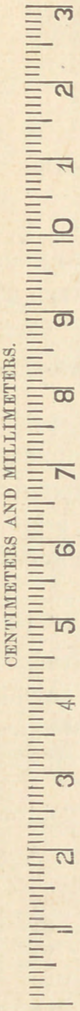

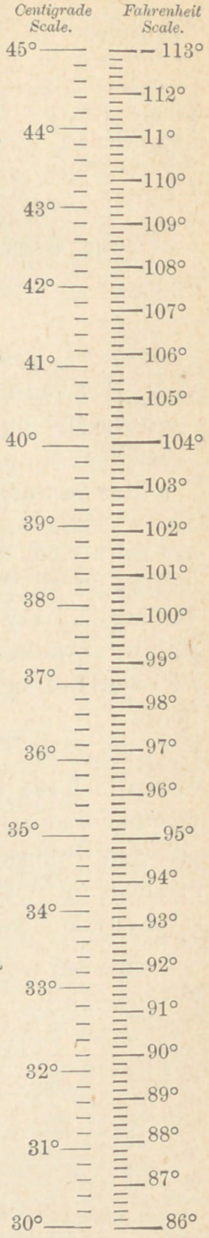

### METRIC FLUID MEASURES.

When using the metric system, fluids are preferably prescribed by weight, employing the gram, its multiples and subdivisions, just the same as with solids, thus avoiding the errors due to refraction, adhesion, and inaccurate measuring vessels. For practical purposes four grams of water may be regarded as equivalent to a fluid drachm of that liquid, and the same may be considered true of tinctures and infusions; syrups, on the average, are about one-third heavier than water, so that a fluid ounce of a syrup will be approximately represented by 43 grams.

If preferred, however, fluids may be prescribed by volume in the metric, just as in the present system, using for that purpose the *Cubic Centimeter*, that is, a volume represented by a cube all of whose sides measure one centimeter. An ordinary back-gammon die is usually about this size. One cubic centimeter (written 1 C. C.) = 16.231 minims. It is approximately regarded as one fourth of a fluid drachm.

### APPROXIMATE EQUIVALENTS OF CUBIC CENTIMETER.

| | | | |
|---|---|---|---|
| 0.001 C. C. | = ......... | 1/65 | minim. |
| 0.01 " | = ......... | 1/6 | " |
| 0.1 " | = ......... | 1 1/2 | " |
| **1. C. C.** | **= ..................** | **15** | **minim.** |
| 4. " | = ......... | 1 | fluid drachm. |
| 16. " | = ......... | 4 | fluid drachms. |
| 32. " | = ......... | 1 | ounce. |

1000 C. C. (usually known as a **Liter**) is a trifle more than one quart, wine measure.

The following prescription—

R: Potassii bromidi, ℥i.
Elixir aurantii, fl., ℥viij.
M.

Would, in metric terms, be written:

Potassic bromide, 32

Orange elixir, 250

### BOOKS AND PAMPHLETS RECEIVED.

A Manual of Physical Diagnosis. By Francis Delafield, M.D., and Ch. F. Stillman, M.D. Cloth, pp. 30. New York: Wm. Wood & Co. Chicago: Jansen, McClurg & Co.

Essentials of Chemistry, Inorganic and Organic, for the Use of Students in Medicine. By B. A. Whithouse, A. M., M.D. Cloth, pp. 257. New York: Wm. Wood & Co. Chicago: Jansen, McClurg & Co.

Clinical Diagnosis; a Handbook for Students and Practitioners of Medicine. Edited by James Finlayson, M.D., with 85 illustrations. Cloth, pp. 546. Philadelphia: H. C. Lea. Chicago: Jansen, McClurg & Co.

The Principles and Practice of Surgery. By J. Ashhurst jr., M.D. Second edition, enlarged and revised, with 542 illustrations. Leather, pp. 1040. Philadelphia: H. C. Lea. Chicago: Jansen, McClurg & Co.

Lectures on Bright's Disease of the Kidney; Delivered at the School of Medicine of Paris. By J. M. Charcot. Collected and published by Drs. Bourneville and Sevestre, Editors of *Le Progrès Médical*, and translated with the permission of the author, by Henry B. Millard, M.D. Cloth, pp. 100. New York: Wm. Wood & Co. Chicago: Jansen, McClurg & Co.

Lectures on Localization in Disease of the Brain. Delivered at the Faculté de Médicine de Paris; 1875. By J. M. Charcot. Edited by Dr. Bourneville. Translated by Ed. P. Fowler, M. D. Cloth, pp. 133. New York: Wm. Wood & Co. Chicago: Jansen, McClurg & Co.

Transactions of the Thirty-third Annual Meeting of the Ohio Medical Society, held at Columbus, May 14, 15, 16, 1878. Columbus, O.

Manual of Prescription Writing; With a Full Explanation of the Methods of Correctly Writing Prescriptions. A Table of Doses expressed in both the Apothecaries' and Metric Systems. Rules for Avoiding Incompatibilities and for Combining Medicines. By M. D. Mann, A.M., M.D. Cloth, pp. 155. New York: G. P. Putnam & Sons. Chicago: Jansen, McClurg & Co.

Practical Surgery, Including Dressing, Bandaging, Ligations and Amputations. By J. Ewing Mears, M.D., with 227 illustrations. Cloth, pp. 279. Philadelphia: Lindsay & Blackiston. Chicago: Jansen, McClurg & Co.

A Handbook of Nursing for Family and General Use. Published under the direction of the Connecticut Training School for Nurses, State Hospital, New Haven, Conn. Cloth, pp. 266. Philadelphia: J. B. Lippincott & Co. Chicago: Jansen, McClurg & Co.

The Pathological Anatomy of the Ear. By Herman Schwartze, M.D., with the Author's Revisions and Additions, and with the Original Illustrations. Translated by J. Orne Greene, A.M., M.D. Cloth, pp. 174. Boston: Haughton, Osgood & Co. Chicago: Jansen, McClurg & Co.

Harvey and His Discovery. By J. M. DaCosta, M. D. Cloth, pp. 57. Philadelphia: J. B. Lippincott & Co. Chicago: Jansen, McClurg & Co.

Transactions of the Pathological Society of Philadelphia. Vol VII. Edited by J. H. C. Simes, M.D. Cloth, pp. 175. Philadelphia: J. B. Lippincott & Co. Chicago: Jansen, McClurg & Co.

A History of the Diagnosis, Pathology and Treatment of Yellow Fever By J. B. Marion, M.D. Reprint from *American Practitioner*, Nov., 1878.

Holden's Human Osteology; Comprising a Description of the Bones, with Delineations of the Attachments of the Muscles, the General and Microscopical Structure of Bone, and its Development. By Luther Holden, F. R. C. S. Fifth edition, revised by the author with the assistance of Alban Doran, F. R. C. S. Cloth, pp. 286. Philadelphia: Lindsay & Blakiston. Chicago: Jansen, McClurg & Co.

The Principles and Practice of Surgery; Being a Treatise on Surgical Diseases and Injuries. By D. Hayes Agnew, M.D., LL. [illegible]. In two volumes. Vol. I. Cloth, pp. 1062. Philadelphia: J. B. Lippincott & Co. Chicago: Jansen, McClurg & Co.

Les Tumeurs Adenoides du Pharynx Nasal, leur Influence sur l'Audition, la Respiration et la Phonation. Leur Traitement. Par le Docteur B. Loewenberg. 1878.

De la Valeur Therapeutic des Courants Continues. Par le Docteur L. J. Teissier, Avec planches, intercallés dans la Texte. 1878.

Annual Report of the Secretary of the Treasury of the State of the Finances for the Year 18[illegible]8.

The Duties of the Profession concerning Prostitution and its Allied Vices. By Fred. H. Gerrish, M.D. Reprint from the Transactions of the (Maine) Association, 1878.

Annual Report of the Surgeon General U. S. Army, 1878.

Diseases of the Nasal Cavity and the Vault of the Pharynx. Translated from the German of Dr. Carl Michel, with an introduction by E. L. Shurley, M.D., and C. C. Yemans, M.D. 1877.

Air and Moisture on Shipboard; A Fragment of Applied Physiology. By Th. J. Turner, A.M., M.D., Ph. D. 1878.

The Constituents of Climate, with Special Reference to the Climate of Florida. By F. D. Lente, A.M., M.D. Reprint from Richmond and Louisville *Medical Journal.*

Report of the Quebec Lunatic Asylum for 1877–8.

Automatic Cerebration as Related to Cerebral Localization. By J. K. Beauduy, M.D. 1878,

Transactions of the Colorado Medical Society, 1877–8.

The Structure of the Colored Blood Corpuscles of Amphiuma Tridactylum, the Frog and Man. By Dr. H. D. Schmidt. Read before the R yal Microscopical Society, April 3, 1878.

The Development of the Nervous Tissues of the Human Embryo. By H. D. Schmidt. Reprint from the *Journal of Nervous and Mental Diseases*, July, 1878.

Case of Repeated Attacks of Appoplexy with Aphasia. By Dr. H. D. Schmidt. Reprint from *Journal of Nervous and Mental Diseases*, July, 1878.

### ANNOUNCEMENTS FOR THE MONTH.

### SOCIETY MEETINGS.

Chicago Medical Society—Mondays, Jan. 13 and 27.

West Chicago Medical Society—Mondays, Jan. 6 and 20.

### CLINICS.

MONDAY.

Eye and Ear Infirmary—2 p. m., Ophthalmological, by Prof. Holmes; 3 p. m., Otological, by Prof. Jones.

Mercy Hospital—1:30 p. m., Surgical, by Prof. Andrews.

Rush Medical College—2 p. m., Dermatological and Venereal, by Dr. Hyde; 3 p. m., Medical, by Dr. Bridge.

Woman's Medical College—2 p. m., Dermatological, by Dr. Maynard.

TUESDAY.

Cook County Hospital—2 to 4 p. m., Medical and Surgical Clinics.

Mercy Hospital—1:30 p. m., Medical, by Prof. Hollister.

WEDNESDAY.

Chicago Medical College—1:30 p. m., Eye and Ear, by Prof. Jones.

Eye and Ear Infirmary—2 p. m., Ophthalmological, by Dr. Hotz.

THURSDAY.

Chicago Medical College—1:30 p. m., Medical, by Prof. Quine.

Rush Medical College—3 p. m., Diseases of the Nervous System, by Prof. Lyman; 4 p. m., Diseases of the Chest, by Prof. Ross.

FRIDAY.

Cook County Hospital—2 to 4 p. m., Medical and Surgical Clinics.

Mercy Hospital—1:30 p. m., Medical, by Prof. Davis.

SATURDAY.

Rush Medical College—2 p. m., Surgical, by Prof. Gunn.

Chicago Medical College—2 p. m., Surgical, by Prof. Isham; 3 p. m., Neurological, by Prof. Jewell.

Woman's Medical College—11 a. m., Ophthalmological, by Dr. Montgomery.

Daily Clinics, from 2 to 4 p. m., at the Central Free Dispensary, and at the South Side Dispensary.

